# Regulating Sodium Deposition Behavior by a Triple‐Gradient Framework for High‐Performance Sodium Metal Batteries

**DOI:** 10.1002/advs.202402321

**Published:** 2024-06-18

**Authors:** Weishan Cao, Mengyue Liu, Weihao Song, Zhen Li, Bingyang Li, Pengfei Wang, Adrian Fisher, Jin Niu, Feng Wang

**Affiliations:** ^1^ State Key Laboratory of Chemical Resource Engineering Laboratory of Electrochemical Process and Technology for Materials Beijing University of Chemical Technology Beijing 100029 P. R. China; ^2^ Beijing Advanced Innovation Center for Soft Matter Science and Engineering Beijing University of Chemical Technology Beijing 100029 P. R. China; ^3^ China Academy of Aerospace Science and Innovation Beijing 100081 P. R. China; ^4^ Department of Chemical Engineering and Biotechnology University of Cambridge New Museums Site, Pembroke Street Cambridge CB2 3RA UK

**Keywords:** carbon framework, gradient, sodium deposition behaviors, sodium metal anode, sodium metal battery

## Abstract

An efficient method for the synthesis of a self‐supporting carbon framework (denoted Gra‐GC‐MoSe_2_) is proposed with a triple‐gradient structure—in sodiophilic sites, pore volume, and electrical conductivity—which facilitates the highly efficient regulation of Na deposition. In situ and ex situ measurements, together with theoretical calculations, reveal that the gradient distribution of Se heteroatoms in MoSe_2_, and its derivatives tailor the sodiophilicity, while the gradient distribution of porous nanostructures homogenizes the Na^+^ diffusion. Therefore, Na deposition occurs from the bottom to the top of the Gra‐GC‐MoSe_2_ framework without dendrite formation. In addition, the gradient in electrical conductivity ensures the stripping process does not lead to dead Na. As a result, a Gra‐GC‐MoSe_2_ modified Na anode (Na@Gra‐GC‐MoSe_2_) shows impressive cycling stability with a high average Coulombic efficiency in an asymmetric cell. In symmetric cells, it also exhibits a long cycling life of 2000 h with a low polarization voltage and works stably even under a large capacity of 10 mAh cm^−2^. Moreover, a Na@Gra‐GC‐MoSe_2_|| Na_3_V_2_(PO_4_)_3_ full cell delivers a high energy density with an excellent cycling performance.

## Graphical abstract

A self‐supporting carbon framework with triple gradients is prepared for high‐performance Na metal anodes without dendrite problems. The tailored gradients of the number of sodiophilic sites, pore volumes, and electrical conductivity homogenize Na^+^ and electron diffusion, thus inducing Na to preferentially deposit on the side furthest away from the separator and enhancing the reversibility of the plating/stripping process.

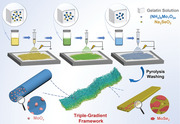

## 1 Introduction

Metallic sodium (Na) is expected to be a promising anode material for low‐cost and high‐energy Na metal batteries due to its highly abundant natural resources, high theoretical capacity (1166 mAh g^−1^) and low working potential (–2.71 V vs standard hydrogen electrode).^[^ ^1^, ^2^ ^]^ However, Na metal anodes (NMAs) suffer from serious safety concerns due to uncontrolled dendrite formation and the infinite dimension change during the Na plating/stripping process, which have hindered its application in practical batteries.^[^ ^3^, ^4^, ^5^, ^6^ ^]^ Over the past few decades, many different approaches have been proposed to overcome the above problems, including optimizing liquid electrolytes,^[^ ^7^, ^8^ ^]^ constructing artificial solid electrolyte interfaces,^[^ ^9^, ^10^, ^11^, ^12^ ^]^ and employing new separators and solid electrolytes.^[^ ^13^, ^14^, ^15^, ^16^, ^17^, ^18^, ^19^ ^]^

**Scheme 1:**
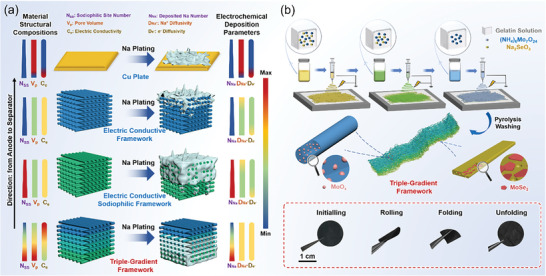
a) Schematic illustration of Na plating on Cu foil, the electrically conducting framework, the electrically conducting sodiophilic framework, and the triple‐gradient framework. b) Schematic illustration of the synthesis of the Gra‐GC‐MoSe_2_ framework and digital photos of the products at various stages.

In particular, 3D hosts have shown promising prospects for fabricating dendrite‐free NMAs. In contrast to traditional current collectors (Cu plates), the electrically conducting framework can reduce the local current density by homogenizing ion/electron fluxes to some extent, which mitigates Na dendrite formation and the infinite volume change.^[^ ^4^, ^20^, ^21^, ^22^, ^23^ ^]^ However, Na deposition occurs preferentially on the top of the framework due to the concentrated electric field and the short Na^+^ pathways for fast ionic diffusion result in the formation of metallic Na near the separator side, leading to the eventual formation of Na dendrites (**Scheme** **1**a).^[^ ^24^ ^]^ In order to induce Na deposition within the framework, different sodiophilic sites (e.g., Ag, Na_2_Te, Na_2_S, g‐C_3_N_4_, etc.) have been introduced into host structures.^[^ ^25^, ^26^, ^27^, ^28^, ^29^, ^30^ ^]^ Although such sodiophilic frameworks enable more internal Na plating, the concentrated electric field and higher Na^+^ diffusivity near the separator side still lead to Na dendrite formation on the framework surface, especially under high plating capacity. Moreover, electrons flow into the anode via the metallic case or tab, which are in direct contact with the framework bottom. The inhomogeneous electron diffusivity within the framework also affects the Na stripping process and can cause dead Na deposits.^[^ ^31^ ^]^ Recently, gradient frameworks with a linear variation in the number of sodiophilic sites have been reported as hosts for NMAs and shown to facilitate gradient Na growth.^[^ ^32^, ^33^ ^]^ Nevertheless, further improvements are required since this strategy cannot tune the electric field distribution and the Na^+^ flux.^[^ ^34^ ^]^ Therefore, the tailored design of the framework with multiple gradients in structure and composition is necessary to further regulate Na deposition behavior by concomitantly tuning the ion/electron diffusivities as well as the number of sodiophilic sites.

In this work, we have presented an efficient method for the synthesis of a self‐supporting carbon framework (denoted Gra‐GC‐MoSe_2_) with a triple‐gradient structure—in sodiophilic sites (Se heteroatoms, MoSe_2_ and its derivatives), pore volume, and electrical conductivity. The number of sodiophilic sites and the pore volume gradually *decrease* from the anode to the separator, thus homogenizing Na^+^ diffusion and inducing Na deposition from the bottom upward. In addition, the electrical conductivity of the framework gradually increases from the bottom to the top. This balances the difference in electron diffusivity and promotes the reversibility of the Na plating/stripping process (Scheme 1a, bottom). Consequently, the triple‐gradient framework effectively induces Na^+^ ions to deposit preferentially at the bottom of the host and inhibits dendrite growth, even under high plating capacity, leading to its good performance in both half‐cells and full cells.

## 2 Results and Discussion

The preparation of the Gra‐GC‐MoSe_2_ framework is illustrated in Scheme 1b (see details in the Supporting Information). Triple gradients were simultaneously realized by a simple electrospinning‐pyrolysis method, which showed higher efficiency than previous strategies for the introduction of multiple‐gradient structures.^[^ ^24^ ^]^ In brief, four aqueous gelatin/(NH_4_)_6_Mo_7_O_24_ solutions with decreasing contents of Na_2_SeO_3_ were used in sequence in an electrostatic spinner. The spun product was then treated by pyrolysis and washed with water to obtain Gra‐GC‐MoSe_2_. This material showed superb flexibility without any damage, even after rolling and folding. The four layers of Gra‐GC‐MoSe_2_ prepared with decreasing doses of Na_2_SeO_3_ are denoted GC@MoSe_2_, GC@MoSe_2_/MoO_x_‐a, GC@MoSe_2_/MoO_x_‐b, and GC@MoO_x_, respectively. Non‐gradient GC@MoSe_2_ and GC@MoO_x_ materials with high thickness were also prepared for comparison.

**Figure 1:**
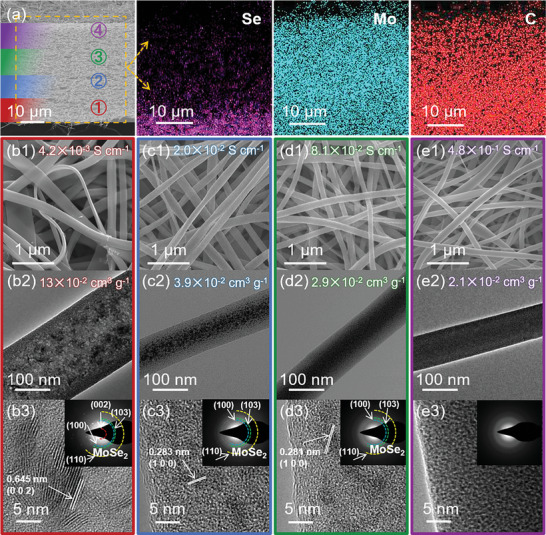
a) Cross‐section SEM and corresponding EDS‐mapping images of Gra‐GC‐MoSe_2_. SEM images (inset: electrical conductivity), TEM images (inset: pore volume), and high‐resolution TEM images (inset: SAED pattern) of (b1–b3) GC@MoSe_2_, (c1–c3) GC@MoSe_2_/MoO_x_‐a, (d1–d3) GC@MoSe_2_/MoO_x_‐b, (e1–e3) GC@MoO_x_. These four samples correspond to layer 1 to layer 4 in Figure a, respectively.

Scanning electron microscopy (SEM) and energy dispersive spectrometry (EDS) were used to characterize the structural composition of the Gra‐GC‐MoSe_2_ framework. As shown in **Figure** **1**a, Gra‐GC‐MoSe_2_ has a relatively loose porous structure with a thickness of ≈45 µm. The EDS mapping images clearly show the gradient in Se content from the bottom to the top, in contrast to the uniform dispersion of Mo and C. X‐ray diffraction (XRD) and Raman spectroscopy (Figure S1, Supporting Information) not only confirm the presence of gelatin‐derived carbon in all four layers but also verify the presence of the MoSe_2_ gradient within the Gra‐GC‐MoSe_2_ framework, suggesting that Na_2_SeO_3_ reacted with (NH_4_)_6_Mo_7_O_24_ and gelatin‐derived carbon to form MoSe_2_. X‐ray photoelectron spectroscopy (XPS) confirmed that Se heteroatoms were doped in the carbon skeleton and contributed to the gradient in Se and MoSe_2_ compositions (Table S2, Supporting Information), while amorphous MoO_x_ exhibited a gradient in the opposite direction (Figure S2, Supporting Information).

In addition to the gradient in chemical composition, the Gra‐GC‐MoSe_2_ framework also exhibits an obvious gradient in morphological structure. High‐resolution SEM images of the four layers of Gra‐GC‐MoSe_2_ (Figure 1b1–e1) show that the structures of the 1D materials change from ultrathin nanobelts (GC@MoSe_2_) to nanofibers (GC@MoO_x_). The electrical conductivity of the four materials gradually increases from bottom to top (Figure 1b1–e1, inset). Transmission electron microscopy (TEM) images confirm the structural changes (Figure 1b2–e2), and the corresponding energy dispersive X‐ray elemental mapping images show well‐dispersed C, N, Se, O, and Mo (Figure S3, Supporting Information), consistent with the XPS results. Moreover, the number of pores and nanoparticles within Gra‐GC‐MoSe_2_ decreases from GC@MoSe_2_ to GC@MoO_x_. N_2_ absorption/desorption isotherms show that both the specific surface area and pore volume gradually decrease from GC@MoSe_2_ to GC@MoO_x_ (Figure S4 and S5 and Table S1, Supporting Information). Specifically, GC@MoSe_2_ has a hierarchical porous structure with a high specific surface area and pore volume of 90.0 and 0.13 cm^3^ g^−1^, while GC@MoO_x_ has a mesoporous structure with a low specific surface area of 13.9 m^2^ g^−1^ and a pore volume 0.021 cm^3^ g^−1^. High‐resolution TEM images further indicate that the morphology of the molybdenum selenides/oxides changes from crystalline MoSe_2_ nanosheets to amorphous MoO_x_ nanoclusters, which is confirmed by the selected area electron diffraction (SAED) patterns (Figure 1b3–e3, inset).

**Figure 2:**
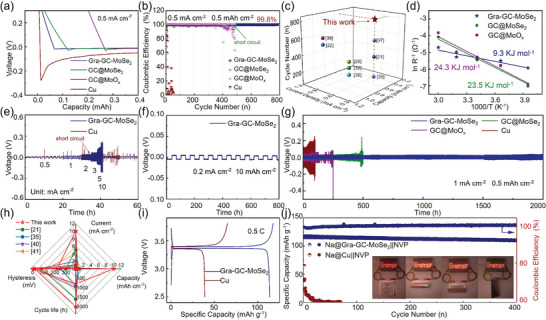
a) Voltage–capacity curves in the initial cycle and b) Coulombic efficiency of Na|Cu, Na|GC@MoO_x_, Na|GC@MoSe_2_ and Na|Gra‐GC‐MoSe_2_ half cells. c) Comparison of the electrochemical performance of the Na|Gra‐GC‐MoSe_2_ half‐cell with other materials reported in the recent literature. d) The activation energy of Na‐ion diffusion within the Gra‐GC‐MoSe_2_, GC@MoSe_2_, and GC@MoO_x_ hosts. Galvanostatic cycling performance of Na|Na symmetric cells using the Gra‐GC‐MoSe_2_ hosts at e) different current densities, f) a current density of 0.2 mA cm^−2^ with a capacity of 10 mAh cm^−2^, and g) a current density of 1 mA cm^−2^ with a capacity of 0.5 mAh cm^−2^ and h) Comparison with other symmetric cells reported in recent literature. i) Capacity–voltage curves of the Na@Gra‐GC‐MoSe_2_||NVP and Na@Cu||NVP full cells. j) Cycling performance of the Na@Gra‐GC‐MoSe_2_||NVP and Na@Cu||NVP full cell. (inset: digital photos of a Na@Gra‐GC‐MoSe_2_||NVP pouch cell powering LED lights in different states.

The Gra‐GC‐MoSe_2_ framework and the other two non‐gradient frameworks (GC@MoSe_2_ and GC@MoO_x_) were then used as hosts for NMAs. Asymmetric cells were first assembled and tested to observe the initial nucleation overpotential of Na on Cu foil and different hosts (**Figure** **2**a). The Na|Gra‐GC‐MoSe_2_ cell exhibited a much smaller initial nucleation overpotential of 19.2 mV than the Na|Cu (278 mV), Na|GC@MoO_x_ (30.7 mV), and Na|GC@MoSe_2_ cells (30.1 mV), indicating that the Na^+^ diffusion and electron transport within the Gra‐GC‐MoSe_2_ framework is faster than in the other three materials.^[^ ^32^ ^]^ Moreover, the Na|Gra‐GC‐MoSe_2_ cell also exhibited the lowest voltage hysteresis (Figure S7, Supporting Information). In addition, the Gra‐GC‐MoSe_2_ cell displayed an impressive cycling stability of 800 cycles with an average coulombic efficiency (CE) of 99.8% at a low current density of 0.5 mA cm^−2^ (Figure 2b), a superior performance to those of the other three cells. Moreover, the Gra‐GC‐MoSe_2_ cell still works stably at higher current densities of 1 and 2 mA cm^−2^ (Figure S6, Supporting Information), highlighting the advantages of the triple‐gradient framework. The high reversibility of Na plating/stripping in the Gra‐GC‐MoSe_2_ framework is superior to previously reported materials prepared using other strategies for NMAs modification (Figure 2c; Table S4, Supporting Information).^[^ ^21^, ^22^, ^28^, ^32^, ^35^, ^36^, ^37^, ^38^ ^]^ By analyzing the temperature‐dependent electrochemical impedance spectra from 253 to 333 K (Figure S9 and Table S3, Supporting Information), the kinetics during Na deposition can be quantitatively investigated based on Arrhenius plots.^[^ ^39^ ^]^ The Na|Gra‐GC‐MoSe_2_ cell has a much smaller activation energy of 9.3 kJ mol^−1^ than the Na|GC@MoSe_2_ (23.5 kJ mol^−1^) and Na|GC@MoO_x_ (24.3 kJ mol^−1^) cells, suggesting that the gradients in pore volume and electrical conductivity in the former material favor the diffusion of both Na^+^ ions and electrons (Figure 2d).

Symmetric cells were subsequently assembled in order to evaluate the galvanostatic cyclic performance of NMAs with gradient and non‐gradient hosts. As shown in Figure 2e, the Gra‐GC‐MoSe_2_ framework enabled the symmetric cell to work steadily with a low overpotential below 220 mV, even at 10 mA cm^−2^. Furthermore, the Gra‐GC‐MoSe_2_ framework afforded an ultrahigh plating/stripping capacity of 10 mAh cm^−2^ over 800 h (Figure 2f). In terms of cycling performance, the symmetric cell using the Gra‐GC‐MoSe_2_ framework showed a long lifetime of over 2000 h with an ultralow voltage hysteresis (Figure 2g), which was superior to previously symmetric cells reported in recent literature (Figure 2h; Table S5, Supporting Information).^[^ ^21^, ^35^, ^40^, ^41^ ^]^ In marked contrast, symmetric cells using GC@MoSe_2,_ GC@MoO_x_, or Cu foil fluctuated obviously and failed to work after a short time (Figure 2g).

In order to evaluate the feasibility of using Gra‐GC‐MoSe_2_‐modified NMAs (denoted Na@Gra‐GC‐MoSe_2_) in practical applications, full cells were assembled by pairing Na@Gra‐GC‐MoSe_2_ with Na_3_V_2_(PO_4_)_3_ (NVP). The N/P ratio of the Na@Gra‐GC‐MoSe_2_||NVP was about ≈3.5. The capacity–voltage curves show that the Na@Gra‐GC‐MoSe_2_||NVP full cell exhibited a large specific capacity of 117.4 mAh g^−1^ and a high initial CE of 98% at 0.5 C, much higher than the corresponding values for the Na||NVP full cell (38.8 mAh g^−1^ and 59%, Figure 2i). A discharge capacity of ≈70 mAh g^−1^ could still be delivered even at 5 C, implying a good rate capability (Figure S10, Supporting Information). In addition, the Na@Gra‐GC‐MoSe_2_||NVP full cell also had a lower hysteresis voltage than the Na||NVP full cell, allowing higher discharge energies. A high energy density of ≈257.0 Wh kg^−1^ can be delivered based on the total weight of Na@Gra‐GC‐MoSe_2_ and NVP, which surpasses by far previously reported values of sodium battery systems with NVP as cathode (Table S6, Supporting Information).^[^ ^42^, ^43^ ^]^ Moreover, the Na@Gra‐GC‐MoSe_2_||NVP full cell exhibited a remarkable cycling performance even with a low N/P ratio (Table S7, Supporting Information), with a capacity retention of ≈95% even after 400 cycles at 0.5 C (Figure 2j), showing its good prospects for practical use. The Na@Gra‐GC‐MoSe_2_||NVP pouch cell could easily power LED lights due to its high energy density and showed good bending performance by virtue of the self‐standing properties of the Gra‐GC‐MoSe_2_ framework (Figure 2j, inset).

**Figure 3:**
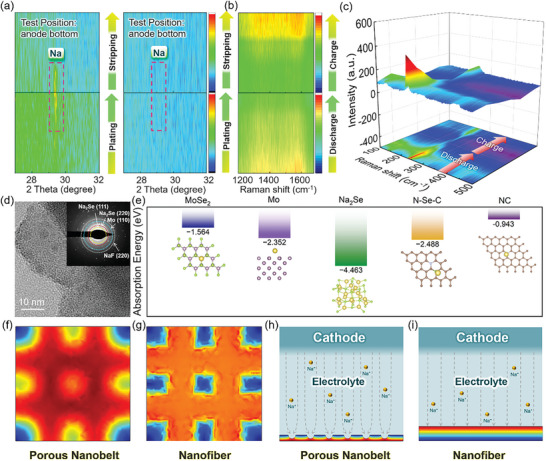
a) In situ XRD patterns of the Gra‐GC‐MoSe_2_ framework (left) and non‐gradient framework (right) in the Na plating/stripping process. b) In situ Raman spectra of carbon within the Gra‐GC‐MoSe_2_ framework in the Na plating/stripping process. c) Ex situ Raman spectra of MoSe_2_ within the Gra‐GC‐MoSe_2_ framework in the Na plating/stripping process. d) High‐resolution TEM image of the Gra‐GC‐MoSe_2_ framework at 0 V (versus Na^+^/Na, inset: SAED pattern). e) Adsorption energies of Na atoms on MoSe_2_, Mo, Na_2_Se, Se,N‐codoped carbon, and N‐doped carbon. COMSOL Multiphysics simulations of the current density distributions for porous nanobelts and nanofibers from f,g) the bottom view and h,i) the side view with the schematic of Na^+^ migration direction.

In situ XRD was employed to detect the plating position of metallic Na within the gradient and non‐gradient hosts (Figure S11, Supporting Information). The XRD patterns for the bottom layer (furthest away from the separator side) of Gra‐GC‐MoSe_2_ and Cu foam during the plating/stripping process are shown in **Figure** **3**a. For the Gra‐GC‐MoSe_2_ framework, the characteristic peak of Na (110) plane was detected during the deposition process but completely disappeared after the stripping process (Figure 3a, left). However, no peak characteristic of Na metal was detected for the non‐gradient host (Figure 3a, right). This confirms that Na is preferentially deposited at the bottom of Gra‐GC‐MoSe_2_ because of the homogeneous Na^+^ flux and sodiophilicity gradient. The complete stripping of Na can be attributed to the uniform and fast electron diffusion within the Gra‐GC‐MoSe_2_ framework resulting from the good structural reversibility of the gelatin‐derived carbon. The high degree of recovery of the D and G bands in the Raman spectra confirms the dynamic stability of the carbon framework during the plating/stripping process (Figure 3b; Figure S12, Supporting Information).

Ex situ Raman spectroscopy highlights the important role of MoSe_2_ and Se heteroatoms in regulating Na deposition (Figure S13, Supporting Information). As shown in Figure 3c, the intensities of the characteristic peaks of MoSe_2_ decreased significantly during the discharging process and partially recovered during the charging process.^[^ ^44^, ^45^, ^46^ ^]^ The TEM image and corresponding SAED pattern of the Gra‐GC‐MoSe_2_ host discharged to 0 V (versus Na^+^/Na) confirm the coexistence of Na_2_Se, Mo, and NaF (Figure 3d). This suggests that most of the MoSe_2_ transformed into Na_2_Se and Mo during the sodiation process and could not revert to MoSe_2_. MoSe_2_ and its derivatives such as Na_2_Se and Mo are all involved in the subsequent plating/stripping process.

DFT calculation results show that MoSe_2_, Mo, and Na_2_Se can spontaneously adsorb Na^+^ ions with adsorption energies of −1.564, −2.352, and −4.463 eV, respectively (Figure 3e). In addition, Se‐doped carbon has a Na^+^ adsorption energy of −2.488 eV, demonstrating that the bottom layer of Gra‐GC‐MoSe_2_ also has good sodiophilicity. Due to the low pore volume of the top layer (GC@MoO_x_), MoO_x_ was embedded into the nanofibers, and the surface N‐doped carbon has a major effect on the Na deposition behavior. The calculated Na^+^ adsorption energy of the N‐doped carbon was only –0.943 eV, showing that the sodiophilicity of GC@MoO_x_ is poorer than that of the underlying layers. As a result of the gradient in sodiophilicity and homogeneous Na^+^/electron diffusivities, Na preferentially nucleated and grew uniformly from the bottom to the top of the Gra‐GC‐MoSe_2_ framework and exhibited high reversibility during the plating/stripping process.

COMSOL Multiphysics simulations were employed to investigate the effect of structure on the current density distributions for Na plating/stripping. Based on their morphological features, nanofibers and porous nanobelts were chosen as the models for GC@MoO_x_ and GC@MoSe_2_, respectively. The color transition from blue to red shown in Figure 3f–i represents the change in relative current density from low to high. From the bottom view, the simulation results show that the centers of the porous nanobelts (Figure 3f) and the edges of the nanofibers (Figure 3g) exhibit the highest current densities. Similar simulation results are shown from the side view in Figure 3h,i. As a result, Na tends to plate on and strip off the surface of the nanofibers, leading to longitudinal Na growth and dendrite formation then becomes inevitable. In contrast, Na preferentially deposits in the pores of the porous nanobelts without dendrite formation. Thus, the gradients in morphology and porosity of Gra‐GC‐MoSe_2_ can facilitate the uniform deposition of dendrite‐free NMAs.

**Figure 4:**
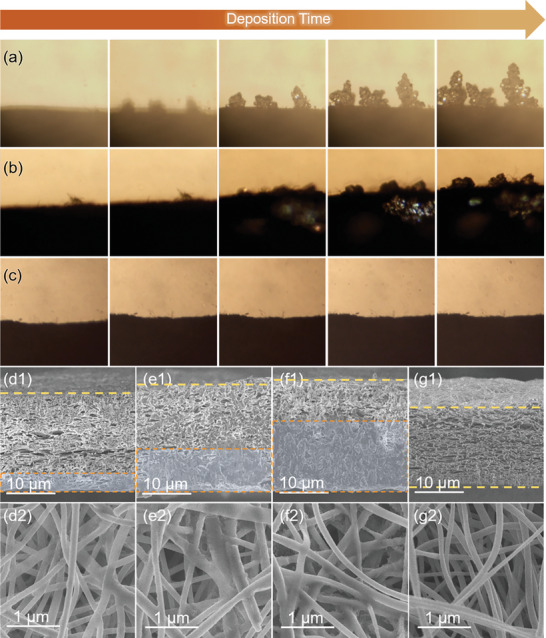
In situ optical microscopy of Na deposition behavior on a) Cu foil, b) GC@MoSe_2_, and c) Gra‐GC‐MoSe_2_ at 0.5 mA cm^−2^ for 4 h. Ex situ cross‐section and surface SEM images of the top (nearest the separator side) for the Gra‐GC‐MoSe_2_ frameworks under Na plating capacities of (d1, d2) 1 mAh cm^−2^, (e1, e2) 2 mAh cm^−2^, (f1, f2) 3 mAh cm^−2^ at 0.5 mA cm^−2^, and (g1, g2) under a Na plating/stripping capacity of 3 mAh cm^−2^ at 0.5 mA cm^−2^.

The modification of NMAs by the Gra‐GC‐MoSe_2_ framework was verified by in situ optical microscopy. As shown in **Figure** **4**a, Na dendrites formed quickly on the Cu surface, causing a large volume change. Although Na dendrites were not observed on the non‐gradient sodiophilic framework (GC@MoSe_2_) in the initial plating process, an obvious volumetric expansion and surface Na dendrite formation were eventually observed due to the uneven ion/electron fluxes (Figure 4b). However, a smooth surface without any dendrites was observed for the Gra‐GC‐MoSe_2_ framework throughout the entire deposition process (Figure 4c), efficiently solving the problems of dendrite formation and infinite expansion of NMAs. In order to characterize the Na deposition behavior more clearly, cross‐section and surface SEM images were obtained for the Gra‐GC‐MoSe_2_ and GC@MoSe_2_ frameworks under different Na plating capacities. During the deposition process, a metallic Na deposit continuously filled the pores from the bottom upward in Gra‐GC‐MoSe_2_ (Figure 4d1–f1). In addition, compacted Na deposits were found at the bottom of Gra‐GC‐MoSe_2_ (Figure S14, Supporting Information), while no deposits were found on the top of the framework (Figure 4d2–f2), proving that the triple‐gradient Gra‐GC‐MoSe_2_ can efficiently regulate Na deposition. In contrast, Na was unevenly plated in the upper layer, with obvious Na deposits on the top of the non‐gradient GC@MoSe_2_ framework (Figure S15, Supporting Information). The digital photos for the bottom side of the Gra‐GC‐MoSe_2_ framework and contrast samples after Na deposition further suggested the unique sodiophilicity of Gra‐GC‐MoSe_2_ (Figure S16, Supporting Information). After the stripping process, all of the Na deposits disappeared without any dead Na remaining and the structure of the Gra‐GC‐MoSe_2_ framework was completely recovered, demonstrating the good reversibility of Na@Gra‐GC‐MoSe_2_ (Figure 4g1, g2).

## 3 Conclusion

We have prepared a self‐standing framework with a triple‐gradient structure by a facile and controllable method. The tailored gradients of the number of sodiophilic sites, pore volumes, and electrical conductivity homogenize Na^+^ and electron diffusion, thus inducing Na to preferentially deposit on the side furthest away from the separator and enhancing the reversibility of the plating/stripping process. Moreover, the porous nanobelt structure provides a large space for Na growth, effectively inhibiting dendrite formation and accommodating anode expansion. Therefore, the modified NMAs exhibited excellent performance in both half‐cells and full‐cells. This work not only provides a new idea for the design of high‐performance NMA hosts but should also promote the practical use of Na metal batteries.

## Conflict of Interest

The authors declare no conflict of interest.

## Supporting information

Supporting Information

## Data Availability

The data that support the findings of this study are available from the corresponding author upon reasonable request.
