# Supplementary material for: Regulating Sodium Deposition Behavior by a Triple‐Gradient Framework for High‐Performance Sodium Metal Batteries
Source: Adv Sci (Weinh). 2024 Jun 18;11(31):2402321. doi: 10.1002/advs.202402321 (PMC11336894; doi:10.1002/advs.202402321)
Supplement: Supplementary file 1 — Supporting Information [file ADVS-11-2402321-s001.docx]

**Supporting Information**

**Regulating sodium deposition behaviors by a triple-gradient framework for high-performance sodium metal batteries**

Weishan Cao,^a,b,#^ Mengyue Liu,^c,#^ Weihao Song,^a,b^ Zhen Li,^c^ Bingyang Li,^c^ Pengfei Wang,^c,^* Adrian Fisher,^d,^* Jin Niu,^a,b,^* Feng Wang ^a,b,^*

^a^State Key Laboratory of Chemical Resource Engineering, Laboratory of Electrochemical Process and Technology for Materials, Beijing University of Chemical Technology, Beijing, 100029, P. R. China

^b^ Beijing Advanced Innovation Center for Soft Matter Science and Engineering, Beijing University of Chemical Technology, Beijing, 100029, P. R. China

^c^ China Academy of Aerospace Science and Innovation, Beijing 100081, P. R. China

^d^ Department of Chemical Engineering and Biotechnology, University of Cambridge, New Museums Site, Pembroke Street, Cambridge, CB2 3RA UK

^#^These authors contributed equally to this work

*Corresponding authors.

E-mail: pengfeiwang@xjtu.edu.cn; [acf42@cam.ac.uk](mailto:acf42@cam.ac.uk); niujin@mail.buct.edu.cn; wangf@mail.buct.edu.cn

**Experimental methods**

**Material preparation**

*Synthesis of Gra-GC-MoSe_2_*: Four parts of gelatin (1.6 g) were mixed with (NH_4_)_6_Mo_7_O_24_·4H_2_O (700 mg) and Na_2_SeO_3_ (700/350/175/0 mg) in deionized water (9 mL) by stirring at 60 °C for 0.5 h, respectively. The four solutions were electrospun in sequence for 1.5 h (ET2535X, Ucalery). The obtained gradient film was then heated at 270 °C for 1 h in air, followed by pyrolysis at 700 °C for 1 h under an argon atmosphere (heating rate: 2.5 °C/min). The pyrolyzed film were washed by water and dried overnight at 60 °C to obtain the Gra-GC-MoSe_2_ framework. The four layers of Gra-GC-MoSe_2_ are named as GC@MoSe_2_, GC@MoSe_2_/MoO_x_-a, and GC@MoSe_2_/MoO_x_-b, GC@MoO_x_ based on the dosage of Na_2_SeO_3_, respectively. Non-gradient GC@MoSe_2_ and GC@MoO_x_ with high thickness were also prepared by increasing the spinning time to 6h using the same procedure. The Gra-GC-MoSe_2_ preparation method was easy to operate, only need to change the amount of Na_2_SeO_3_ to realize the gradient structure. Furthermore, each layer of Gra-GC-MoSe_2_ thickness could be flexibly adjusted by only controlling the electrostatic spinning time, which was more convenient than the currently reported gradient structure of composites.

**Material characterization**

The morphology and chemical composition of the samples were investigated by SEM (JEOL, FE-JSM-6701F) and field-emission TEM (FEI, FEI Tecnai G2F30) with EDX and SAED. The crystal phase was analyzed by XRD on an Ultima IV X-Ray Diffractometer with Ni filtered Cu Kα radiation (λ = 1.5406 Å) at a voltage of 40 kV and a current of 40 mA. The surface chemical states of the samples were examined by an ESCALAB 250 XPS system with an Al Kα radiation source. Raman spectrum was collected through a LabRam HR800 using the laser excitation source at 633 nm. The porosities of the samples were analyzed based on nitrogen adsorption–desorption isotherms (Quantachrome AUTOSORB-1). The pore size distributions of the samples were evaluated by a nonlocal density functional theory method. Electric conductivities of the samples were measured by a four-probe resistivity tester (RTS-8).

**Electrochemical measurements**

For the preparation of NVP cathodes, the mass ratios of NVP, PVDF, and carbon black were controlled at 8:1:1. They were mixed in N-methyl-2-pyrrolidone (NMP) solvent under constant grinding and stirring to form a homogeneous slurry, and then the cathode electrode was obtained by coating the slurry onto Al foil by doctor blading, followed by vacuum drying at 120 °C for 12 h. The NVP loading is ~2.5 mg cm^-2^.

CR2032 coin cell and pouch cell were assembled in an Ar-filled glovebox to evaluate the half-cell and full-cell performance, respectively. The adopted electrolyte was 1 M NaPF_6_ in diglyme. The electrolyte amount for the half cell and full cell were 200 μL and ~ 10 μL mg^-1^ (based on the weight of electrode materials), respectively. Glass fiber membrane was used as the separator. For half cells, hosts and collectors were cut into round pieces with diameter of 14 mm, and the Na foil diameter was also controlled at 14 mm. The weight of the Gra-GC-MoSe_2_ host was ~0.50 mg cm^-2^.

The assembled cells were tested in a LAND multichannel battery test system. The EIS was performed using a CHI660E electrochemical workstation with a frequency range from 100 kHz to 0.01 Hz. Na plating/stripping tests were conducted by the galvanostatic charge–discharge experiments using Na|Cu or Na|host and Na|Na or Na@host|Na@host half cells. For Na|Cu or Na|host half cells, Na foil and Cu foil or host were matched to evaluate the cycling stability and CE of Cu foil or host. For Na|Na or Na@host|Na@host half cells, a pair of identical electrodes were matched to evaluate the cycling stability and voltage fluctuation of the Na anodes at different current density with a capacity of 0.5 mAh cm^–2^ after a pre-electrodeposition process with a Na plating capacity of 2 mAh cm^-2^. The full cells were cycled at a constant rate within the voltage range of 2.5 to 3.8 V (1 C = 118 mA g^-1^). The energy density E (Wh kg^–1^) and power density P (W kg^–1^) were calculated using the following equations:

$E=\int IVdt=\frac{E^{'}}{m}$ (1)

$P=\frac{E}{t}$ (2)

where *I* is the current density (A kg^–1^), *V* is the voltage (V), and *t* is the discharge time (h). $E^{'}$ is the energy (Wh), m is the weight based on the total mass of the host, deposited Na, and NVP material (kg). For the Na@Gra-GC-MoSe_2_||NVP full cell, the total mass of the host and deposited Na is ~2.202 mg, the mass of NVP material is ~4.043 mg. The N/P ratio of the Na@Gra-GC-MoSe_2_||NVP was about ~3.5. The thickness of the cathode and anode materials are ~45 μm and ~8 μm, respectively.

$$E=\frac{1.605 mWh}{\left( 2.202+4.043 \right)\times{10}^{-6} kg}=257.0 Wh {kg}^{-1}$$

$$P=\frac{E}{t}=\frac{257.0 \mathrm{Wh}\mathrm{kg}^{-1}}{2.100 h}=122.4 W {kg}^{-1}$$

$$E_{V}=\frac{1.605 mWh}{3.141\times{7.000}^{2}\times\left( 45.00+8.000 \right)\times{10}^{-3}\times{10}^{-6} L}=196.8 Wh L^{-1}$$

The *in situ* optical microscopy was conducted on an optical microscope (CIS-OM-002) with an electrolytic cell (Beijing Science Star Technology). Gra-GC-MoSe_2_, GC@MoSe_2_, and Cu foil were used as the working electrodes and Na foil was used as the counter electrode. The evolution of the Na deposition could be clearly observed on the working electrode side at a constant current density of 0.5 mA cm^–2^.

**DFT calculations/Finite-element Simulation**

The calculations were performed by the Material Studio software package, the exchange-correlation energy was approximately described by the Perdew-Burke-Ernzerhof (PBE) functional based on the generalized gradient approximation (GGA). The convergence criteria for the ionic relaxation and the electronic self-consistent calculation were set to 0.02 eV Å^-1^ and 10^-5^ eV. The Brillouin zone integration was performed with a 3 × 3 × 1 Monkhorst-Pack k-points mesh. The long-range dispersion correction for the van der Waals interaction was implemented through the DFT-D3 method in all calculations.^[S1]^

Simulations of the current density distributions in this work were carried out using COMSOL Multiphysics 5.4 software. The physical modulus adopted was “tertiary current distribution”. The Na^+^ deposition process was simulated by coupling the Nernst–Planck equation with the Butler–Volmer equation. Two-dimensional and three-dimensional models were first established to display the entire geometry construction consisting of two electrodes, occupied by the bulk electrolyte. It should be noted that this module can only establish an ideal system due to the model simplification for the calculation. The Na ion conductivity of the electrolyte was set as 0.5 S m^–1^, the initial concentration was 1 M, the diffusion coefficient of Na^+^ was 1.6 × 10^–10^.^[S2]^ To acquire high-quality calculation results, the simulation model is built by using ultrafine grid division.


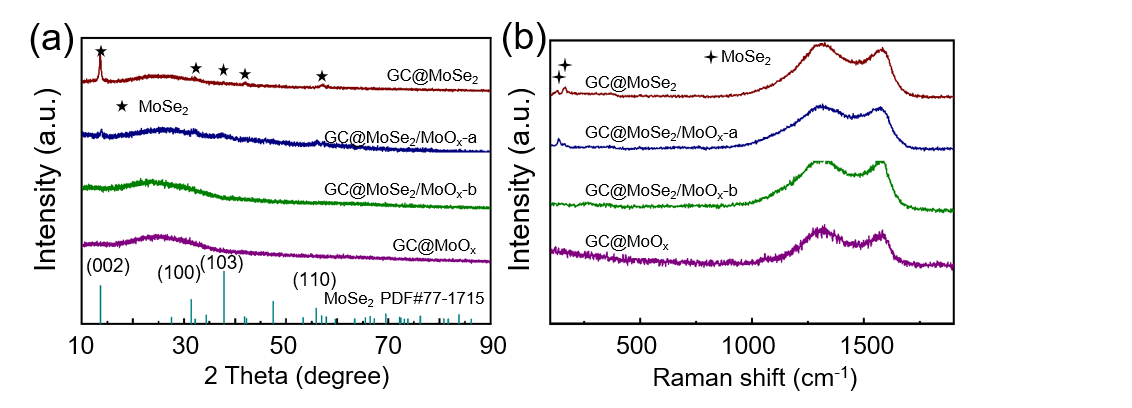


Figure S1. (a) XRD patterns and (b) Raman spectra of GC@MoSe_2_, GC@MoSe_2_/MoO_x_-a, GC@MoSe_2_/MoO_x_-b, and GC@MoO_x_.

The XRD patterns and Raman spectra of four films (GC@MoSe_2_, GC@MoSe_2_/MoO_x_-a, GC@MoSe_2_/MoO_x_-b and GC@MoO_x_) are shown in Figure S1. As shown in Figure S1a, the broad and weak peaks centered at ~26º are attributed to the characteristic reflection of graphitic (002) plane, confirming the existence of gelatin-derived carbon in all the samples. In addition, characteristic peaks with decreasing peak intensity of MoSe_2_ are observed in GC@MoSe_2_, GC@MoSe_2_/MoO_x_-a, GC@MoSe_2_/MoO_x_-b, confirming the formation of MoSe_2_ derived from Na_2_SeO_3_ and (NH_4_)_6_Mo_7_O_24_·4H_2_O. The existence of the MoSe_2_ and gelatin-derived carbons are also verified by the Raman spectra which exhibit characteristic bands of MoSe_2_ and G/D bands of carbon, respectively (Figure S1b). No obvious peaks are observed in the XRD pattern and Raman spectrum of GC@MoO_x_, indicating the amorphous structure of the Mo compounds in GC@MoO_x_.


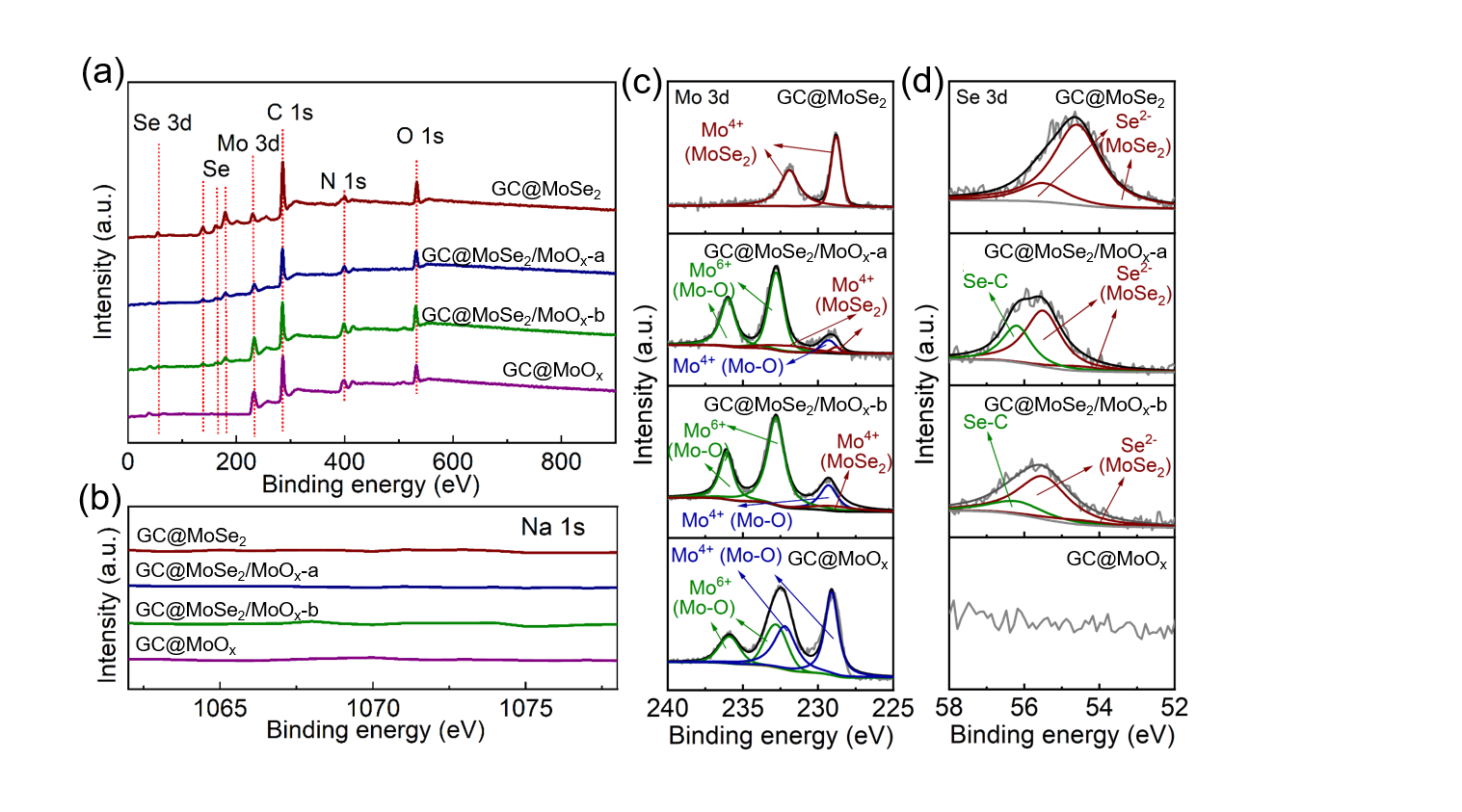


Figure S2. (a) XPS spectra, (b) Na 1s XPS spectra, and high-resolution (c) Mo 3d, (d) Se 3d XPS spectra of GC@MoSe_2_, GC@MoSe_2_/MoO_x_-a, GC@MoSe_2_/MoO_x_-b, and GC@MoO_x_.

The elemental composition and states of the four samples were analyzed by XPS. As shown in Figure S2a, obvious Mo, Se, C, N, O species are shown in the XPS spectra of GC@MoSe_2_, GC@MoSe_2_/MoO_x_-a, GC@MoSe_2_/MoO_x_-b, revealing the existence of the selenides/oxides of molybdenum and Se,N-doping in the gelatin-derived carbons. Obvious Mo, C, N, O species are shown in the XPS spectra of GC@ MoO_x_, revealing the existence of the oxides of molybdenum and N-doping in the gelatin-derived carbons. As shown in Figure S2b, there is no peak around 1072 eV which is the binding energy of Na 1s, indicating the complete removal of sodium compounds from the host. As shown in Figure S2c, the Mo 3d XPS spectrum of GC@MoSe_2_ indicates that Mo exists in the form of Mo^4+^ (MoSe_2_), confirming the MoSe_2_ in GC@MoSe_2_. The Mo 3d XPS spectra of GC@MoSe_2_/MoO_x_-a and GC@MoSe_2_/MoO_x_-b show that Mo exists in the form of Mo^4+^ (MoSe_2_, MoO_x_) and Mo^6+^ (MoO_x_), confirming the existence of MoO_x_ and MoSe_2_ both in GC@MoSe_2_/MoO_x_-a and GC@MoSe_2_/MoO_x_-b. The Mo 3d XPS spectrum of GC@MoO_x_ demonstrates that Mo exists in the form of Mo^6+^ (MoO_x_) and Mo^4+^ (MoO_x_), confirming the existence of MoO_x_ in GC@MoO_x_.^[S3-S8]^ In the Se 3d XPS spectra (Figure S2d), the GC@MoSe_2_, GC@MoSe_2_/MoO_x_-a and GC@MoSe_2_/MoO_x_-b samples all shows two typical peaks located at 54.6 and 55.5 eV, which can be attributed to the Se 3d_5/2_ and Se 3d_3/2_ orbitals of Se^2−^ species in MoSe_2_, respectively.^[S7-S10]^ Besides, GC@MoSe_2_/MoO_x_-a and GC@MoSe_2_/MoO_x_-b exhibit peaks at 56.2 eV which can be assigned to the Se-C bonds formed between MoSe_2_ and carbon.^[S10, S11]^


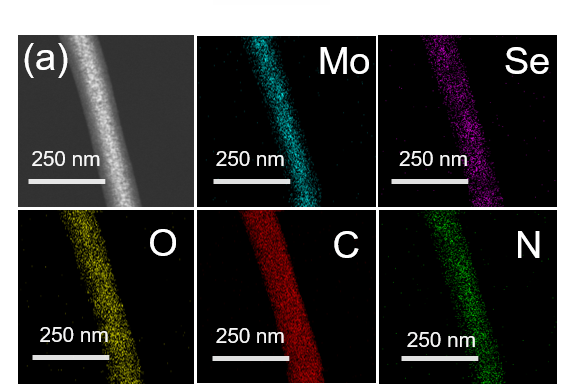


Figure S3. EDX-mapping image of the GC@MoSe_2_/MoO_x_-b framework.


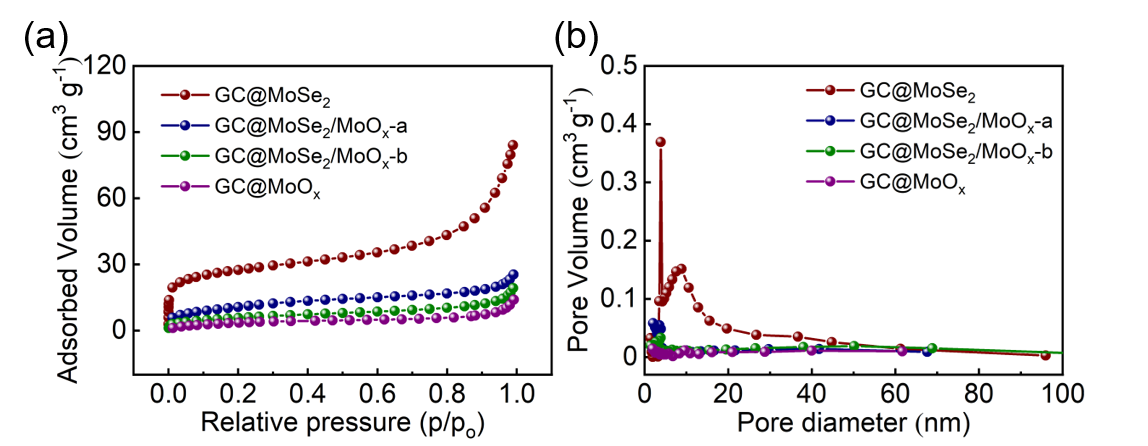


Figure S4. (a) N_2_ absorption/desorption isotherm plots and (b) pore-diameter distribution plots of GC@MoSe_2_, GC@MoSe_2_/MoO_x_-a, GC@MoSe_2_/MoO_x_-b, and GC@MoO_x_.


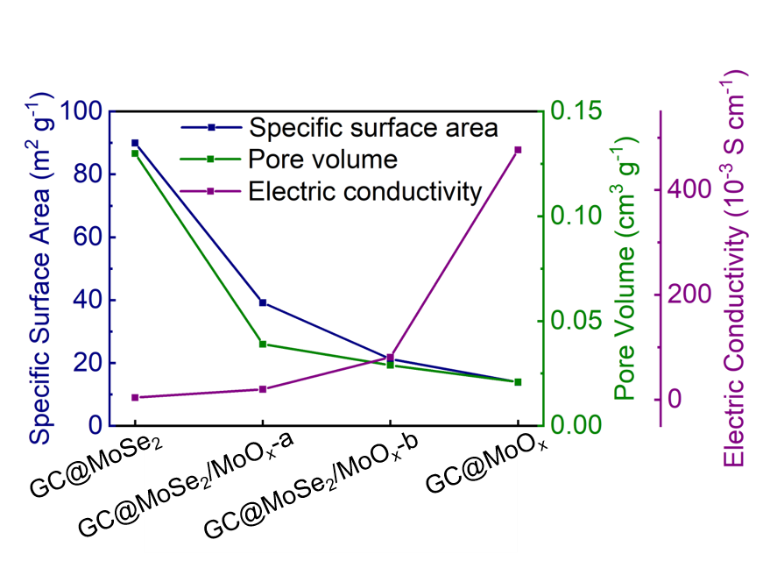


Figure S5. Specific surface area, pore volume, and electric conductivity of GC@MoSe_2_, GC@MoSe_2_/MoO_x_-a, GC@MoSe_2_/MoO_x_-b, and GC@MoO_x_.


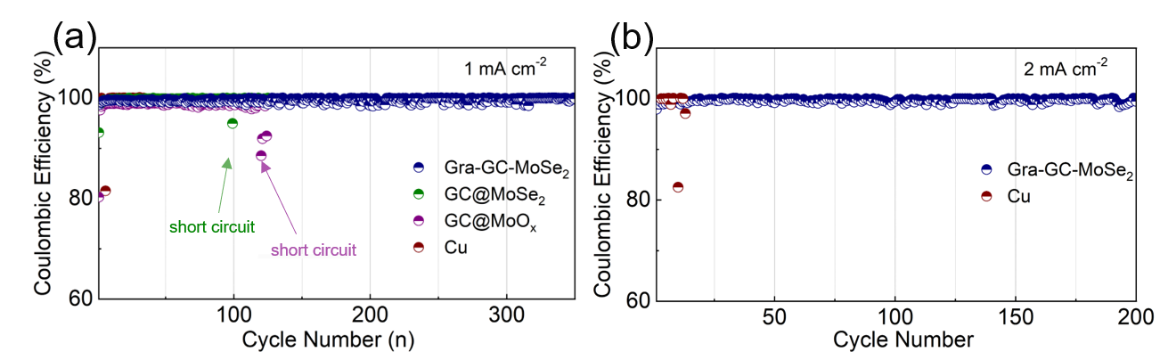


Figure S6. Coulombic efficiency of Na|Cu, Na|GC@MoO_x_, Na|GC@MoSe_2_, and Na|Gra-GC-MoSe_2_ half cells at current density of 1 mA cm^–2^ and 2 mA cm^–2^ with a capacity of 0.5 mAh cm^–2^.


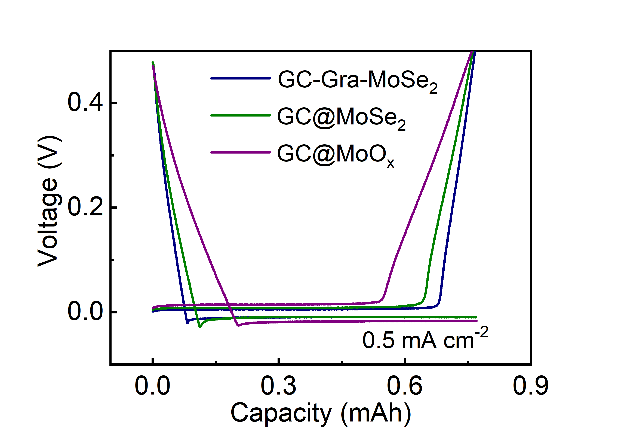
Figure S7. The voltage-capacity curves of Na plating/stripping on Gra-GC-MoSe_2_, GC@MoSe_2_ and GC@MoO_x_ frameworks at current density of 0.5 mA cm^–2^ with a capacity of 0.5 mAh cm^–2^.


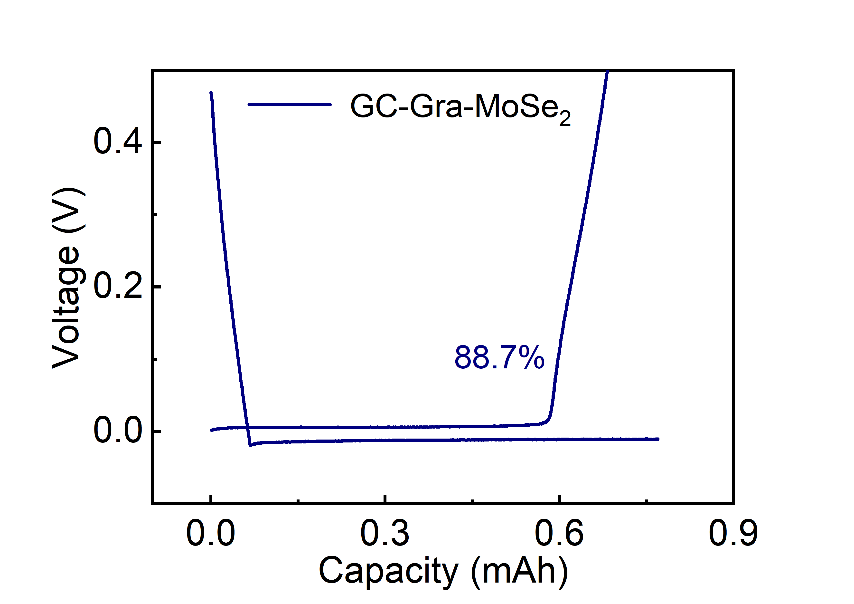


Figure S8. The initial CE of Gra-GC-MoSe_2_ in half cell.


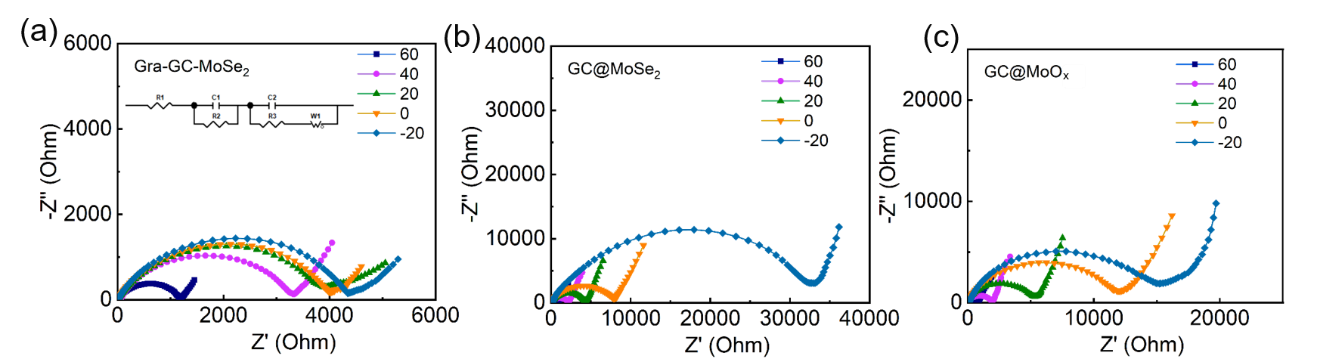


Figure S9. Nyquist plots at different temperatures of the (a) Na|Gra-GC-MoSe_2_ (inset: equivalent-circuit diagram), (b) Na|GC@MoSe_2_, and (c) Na|GC@MoO_x_ half cells.


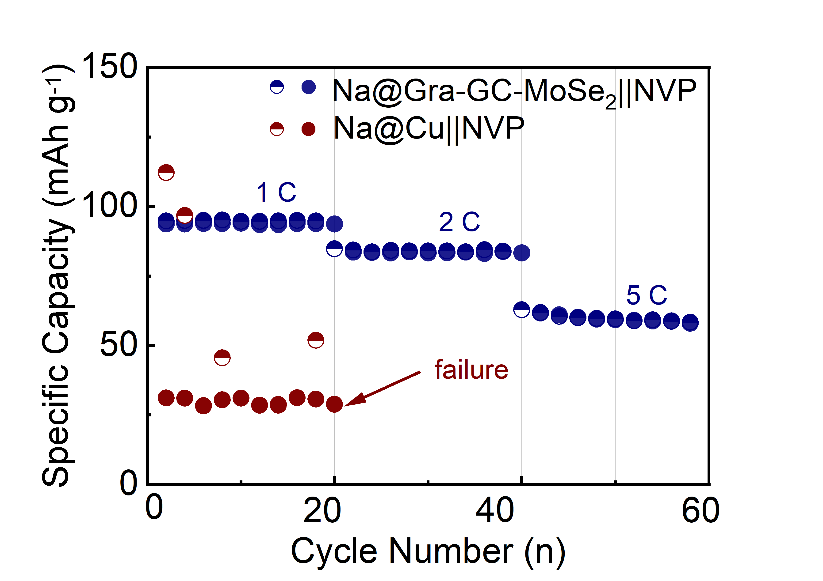


Figure S10. The rate performances of the Na@Gra-GC-MoSe_2_||NVP and Na@Cu||NVP full cells.


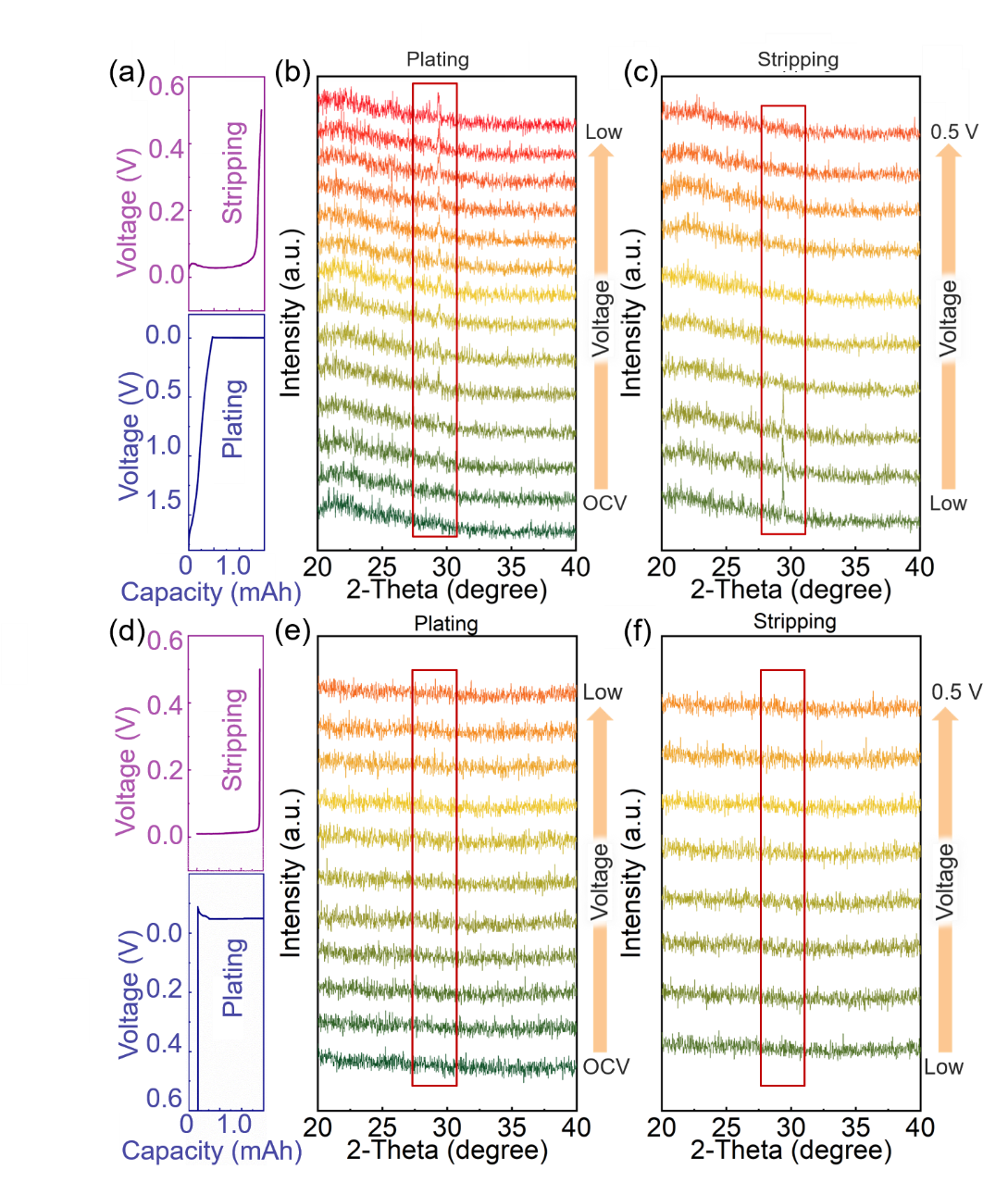


Figure S11. *In situ* XRD patterns of (a-c) the Gra-GC-MoSe_2_ framework and (d-f) Cu foam in the Na plating/stripping process.


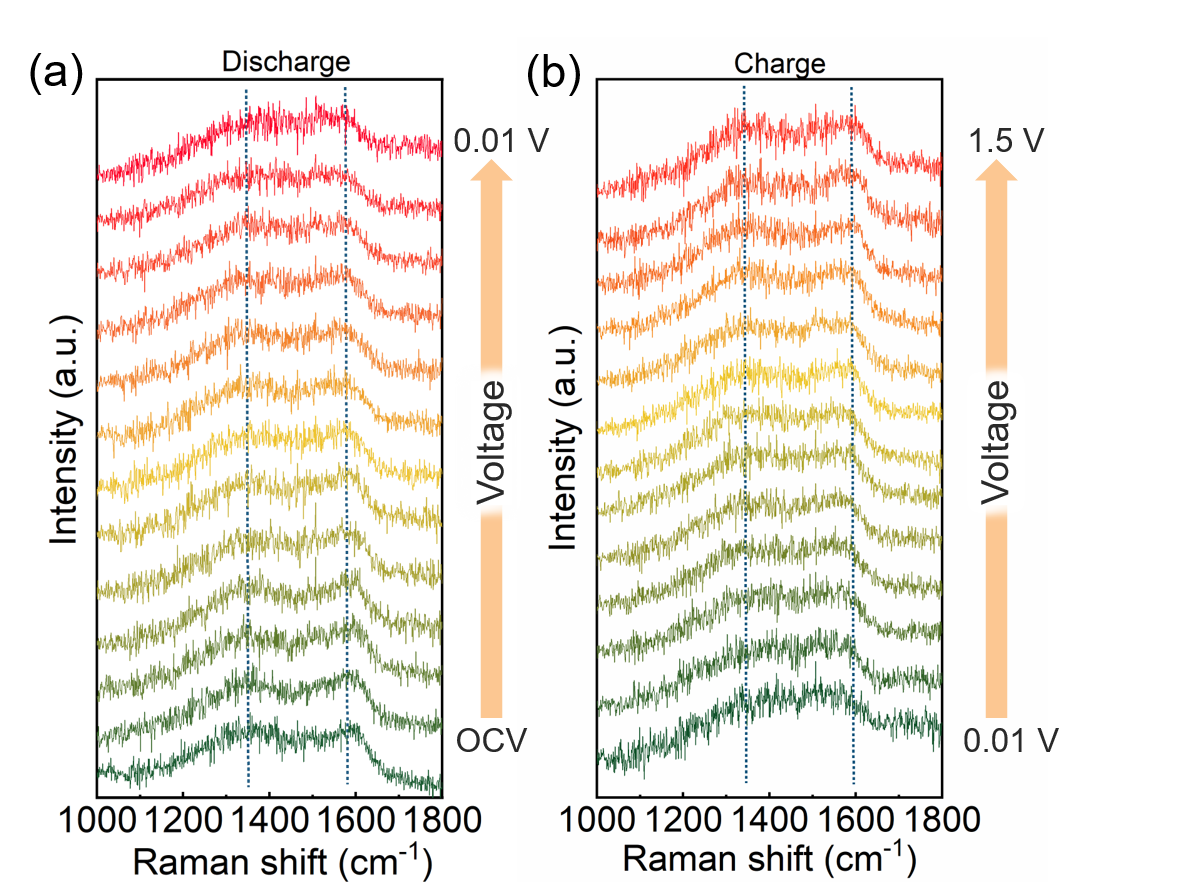


Figure S12. *In situ* Raman spectra of carbon within the Gra-GC-MoSe_2_ framework in the Na plating/stripping process.


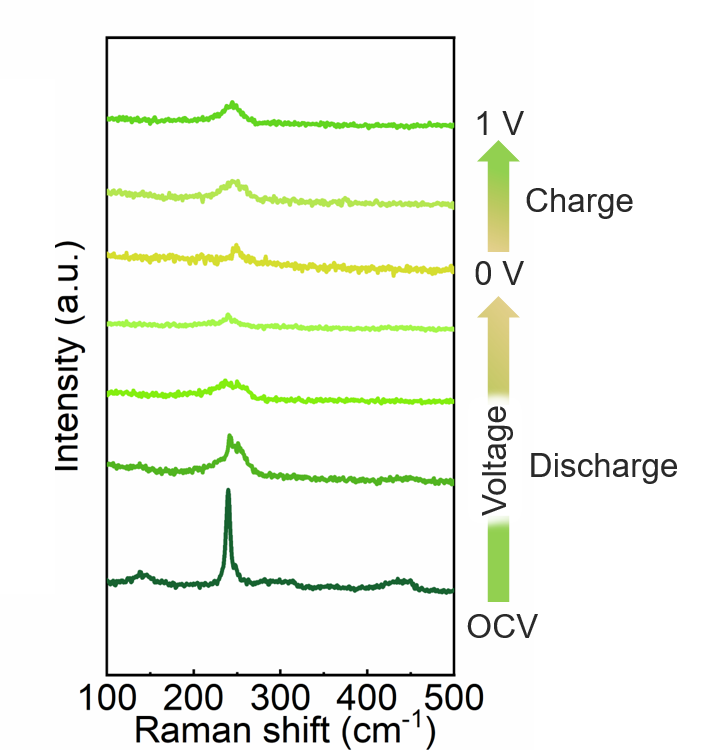


Figure S13. *Ex situ* Raman spectra of MoSe_2_ within the Gra-GC-MoSe_2_ framework in the Na plating/stripping process.


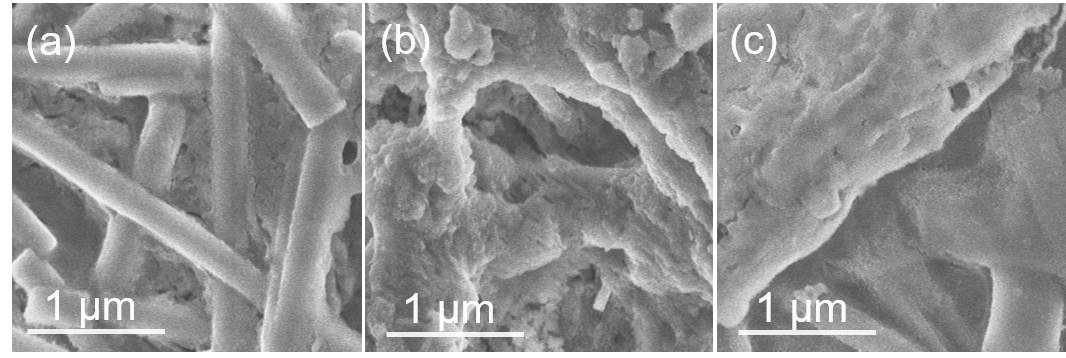


Figure S14. SEM images of the bottom (away from separator side) for the Gra-GC-MoSe_2_ frameworks under Na plating capacities of (a) 1 mAh cm^–2^, (b) 2 mAh cm^–2^, (c) 3 mAh cm^–2^ at 0.5 mA cm^–2^.


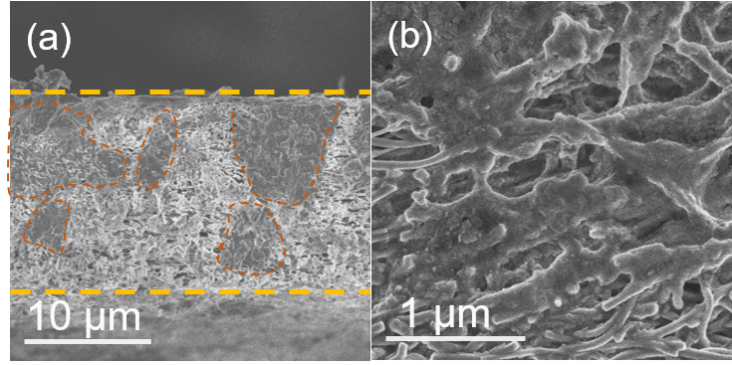


Figure S15. (a) Cross-section and (b) surface (near separator side) SEM images of the GC@MoSe_2_ host under a Na plating capacity of 3 mAh cm^–2^ at 0.5 mA cm^–2^.


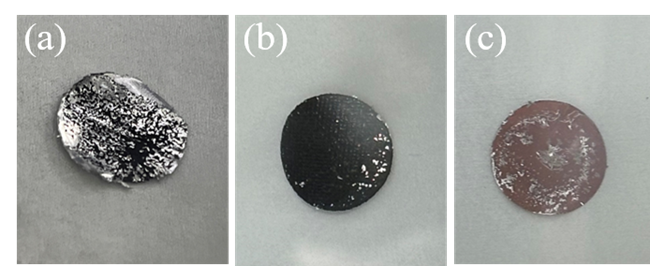


Figure S16. Digital photos of the bottom side of the Gra-GC-MoSe_2_ framework, carbon cloth, and Cu foil after Na deposition.

Table S1. Pore structure parameters of GC@MoSe_2_, GC@MoSe_2_/MoOx-a, GC@MoSe_2_/MoO_x_-b, and GC@MoO_x_.

|  |  |  | specific surface area^c^ (m^2^ g^-1^) | |
| --- | --- | --- | --- | --- |
| Samples | *S*_BET_^a^ (m^2^ g^-1^) | *V*_t_^b^ (cm^3^ g^-1^) | micropore | mesopore |
| GC@MoSe_2_ | 90.0 | 0.130 | 44.2 | 45.7 |
| GC@MoSe_2_/MoO_x_-a | 39.2 | 0.039 | 0 | 39.2 |
| GC@MoSe_2_/MoO_x_-b | 21.3 | 0.029 | 0 | 19.1 |
| GC@MoO_x_ | 13.9 | 0.021 | 0 | 13.9 |

^a^Total specific surface area, calculated by the BET method;

^b^Total pore volume (p/p_0_ = 0.99);

^c^Specific surface area of micropores and mesopores/macropores, calculated by the t-plot method.

Table S2. The atomic content of Se and corresponding MoSe_2_ content in different frameworks.

| Samples | Se (at %) | MoSe_2_ (at %) |
| --- | --- | --- |
| GC@MoSe_2_ | 2.18 | 1.09 |
| GC@MoSe_2_/MoO_x_-a | 1.18 | 0.59 |
| GC@MoSe_2_/MoO_x_-b | 0.94 | 0.47 |
| GC@MoO_x_ | 0 | 0 |

Table S3. The specific impedance values of the Na|Gra-GC-MoSe_2_, Na|GC@MoSe_2_ and Na|GC@MoO_x_ half cells.

| Test temperature (°C) | The specific impedance values (Ω) | | | |
| --- | --- | --- | --- | --- |
|  | Na\|Gra-GC-MoSe_2_ | Na\|GC@MoSe_2_ | Na\|GC@MoO_x_ | |
| 60 | 110.4 | 59.2 | | 45.8 |
| 40 | 195.5 | 144.3 | | 146.5 |
| 20 | 219.3 | 231.5 | | 196.7 |
| 0 | 245.0 | 326.0 | | 326.0 |
| -20 | 376.8 | 1117.0 | | 989.5 |

Table S4. Comparison of the electrochemical performance of Na|Gra-GC-MoSe_2_ half-cell with other half cells reported in recent literatures.

| Na half- cell | Current  (mA cm^−2^) | Cycle  Number (n) | Ref.^a^ | |  |
| --- | --- | --- | --- | --- | --- |
| **Gra-GC-MoSe_2_** | **0.5** | **800** | | **This work** | |
| Sn_4_P_3_ NPs@CNF | 1 | 520 | | [22] | |
| Sb@HPCNF | 0.5 | 330 | | [32] | |
| MgF_2_@RGO | 0.5 | 350 | | [21] | |
| FEIE | 0.5 | 100 | | [35] | |
| Cu-Cu@C | 1 | 600 | | [36] | |
| Ti_3_C_2_ MXene@g-C_3_N_4_ | 0.5 | 400 | | [28] | |
| mPG-12@PP | 0.5 | 550 | | [37] | |
| SFC/CNTs@Cu | 0.5 | 250 | | [38] | |

^a^The reference numbers are corresponding to those in the manuscript.

Table S5. Comparison of the cycling performance of Na|Na symmetric cells using the Gra-GC-MoSe_2_ hosts with other symmetric cells reported in recent literatures.

| Electrode | Current  (mA cm^−2^) | Capacity  (mAh cm^−2^) | Cycle Life  (h) | Hysteresis  (mV) | Ref.^a^ | |  |
| --- | --- | --- | --- | --- | --- | --- | --- |
| **Na@**  **Gra-GC-MoSe_2_** | **0.2** | **10** | **800** | **6** | | **This work** | |
|  | **1** | **0.5** | **2000** | **18** | |  |  |
|  | **10** | **0.5** | **1** | **220** | |  |  |
| Na/L700 | 1 | 0.5 | 1000 | 25 | | [40] | |
|  | 2 | 0.5 | 10 | 50 | |  |  |
| Na/SnSe | 1 | 1 | 900 | 50 | | [41] | |
|  | 4 | 1 | 6 | 120 | |  |  |
| MgF_2_@RGO | 0.5 | 0.5 | 1600 | 80 | | [21] | |
|  | 5 | 0.5 | 2 | 300 | |  |  |
| FEIE | 1 | 1 | 900 | 250 | | [35] | |

^a^The reference numbers are corresponding to those in the manuscript.

Table S6. Comparison of energy densities with previously reported values.

| Anode materials | Cathode | power density  (W kg^-1^) | Energy density  (W h kg^-1^) | Ref.^a^ |
| --- | --- | --- | --- | --- |
| **Gra-GC-MoSe_2_** | **NVP** | **122.4** | **257.0** | **This work** |
| A-SnS–G@Na | NVP | / | 118 | [42] |
| Bi_0.67_NbS_2_ | NVP | 234 | 183 | [43] |

^a^The reference numbers are corresponding to those in the manuscript.

Table S7. Comparison of the N/P ratio and performance of the Na@Gra-GC-MoSe_2_||NVP full cell with other full cells reported in recent literatures

| Full cell | N/P ratio | Cycle number | Capacity retention | | Ref. |  |  |
| --- | --- | --- | --- | --- | --- | --- | --- |
| This work | | 3.5 | 400 | 95% |  | | |
| Na\|\|NVPF | | 4 | 250 | 87% | S12 | | |
| Na\|\|NVPF | | 4 | 220 | 90% | S13 | | |
| Na\|Co-NWS\|NVP | | 7 | 170 | 88% | S14 | | |

**References**

[S1] D. Zhang, W. Song, L. Lv, C. Gao, F. Gao, H. Guo, R. Diao, W. Dai, J. Niu, X. Chen, J. Wei, M. Terrones, Y. Wang, *Carbon* **2023**, 214, 118315.

[S2] C. Bao, J. Wang, B. Wang, J. Sun, L. He, Z. Pan, Y. Jiang, D. Wang, X. Liu, S. X. Dou, J. Wang, *ACS Nano* **2022**, 16, 17197.

[S3] F. Yang, N. J. Libretto, M. R. Komarneni, W. Zhou, J. T. Miller, X. Zhu, D. E. Resasco, *ACS Catal.* **2019**, 9, 7791.

[S4] X. Han, C. Wu, H. Li, Y. Zhang, W. Sun, B. Jia, I. D. Gates, Z. H. Huang, T. Ma, *Adv. Funct. Mater.* **2022**, 32, 2113209.

[S5] B. Li, W. Cao, S. Wang, Z. Cao, Y. Shi, J. Niu, F. Wang, *Adv. Sci.* **2022**, 9, 2204232.

[S6] J. Wang, Y. Shao, F. Yuan, H. Sun, D. Zhang, Z. Li, S. Ramesh, H. J. Woo, B. Wang, *J. Energy Chem.* **2023**, 80, 291.

[S7] H. Liu, H. Guo, B. Liu, M. Liang, Z. Lv, K. R. Adair, X. Sun, *Adv. Funct. Mater.* **2018**, 28, 1707480.

[S8] C. Liu, K. Wang, X. Zheng, X. Liu, Q. Liang, Z. Chen, *Carbon* **2018**, 139, 1.

[S9] Y. Hoe Seon, Y. Chan Kang, J. S. Cho, *Chem. Eng. J.* **2021**, 425, 129051.

[S10] L. Meng, Y. Yao, J. Liu, Z. Wang, D. Qian, L. Zheng, B.-L. Su, H.-E. Wang, *J. Energy Chem.* **2020**, 47, 241.

[S11] W. Dong, C. Li, C. Wang, L. Wu, Z. Hu, J. Liu, L. Chen, Y. Li, B. Su, *Small* **2022**, 18, 2105169.

[S12] Y. Wang, H. Yang, J. Xu, P. Tang, Q. Wei, T. Hu, X. Gao, Z. Guo, R. Fang, G. Hu, S. Bai, F. Li, *J. Am. Chem. Soc.* **2024**, 146, 7332.

[S13] J. Chen, Y. Peng, Y. Yin, M. Liu, Z. Fang, Y. Xie, B. Chen, Y. Cao, L. Xing, J. Huang, Y. Wang, X. Dong, Y. Xia, *Energy Environ. Sci.* **2022**, 15, 3360.

[S14] H. Liu, X. Zheng, Y. Du, M. C. Borrás, K. Wu, K. Konstantinov, W. K. Pang, S. Chou, H. Liu, S. Dou, C. Wu, *Adv. Mater.* **2023**, 36, 2307645.
